# Maternal mental health: Women’s voices and data from across the globe

**DOI:** 10.1186/s12884-022-05064-5

**Published:** 2022-10-28

**Authors:** Maggie Redshaw, Karen Wynter

**Affiliations:** 1 National Perinatal Epidemiology Unit, Nuffield Department of Population Health, University of Oxford, Richard Doll Building, Old Road Campus, OX3 7LF Oxford, UK; 2 Brazelton Centre UK, CIO, 66 Devonshire Road, CB1 2BL Cambridge, UK; 3 School of Nursing and Midwifery, Deakin University, 1 Gheringhap St, 3220 Geelong, VIC Australia; 4 The Centre for Quality and Patient Safety Research in the Institute for Health Transformation, Deakin University – Western Health Partnership, Western Health, 176 Furlong Road, 3021 St Albans, VIC Australia

Pregnancy and childbirth represent major life events for women, their families and communities. *BMC Pregnancy and Childbirth* publishes many papers concerning women’s health in relation to these events, associated clinical interventions, the organisation of maternity care and differences between groups and outcomes. For this Collection however, the focus has been specifically on women’s mental health during pregnancy and the first postnatal year.

## Rationale for this collection

The emotional, personal and societal costs of poor mental health are substantial [1]. The many ways in which positive and poor perinatal mental health can be explored and evidenced is shown in the diverse papers making up the Maternal Mental Health Collection, recently published in *BMC Pregnancy and Childbirth*. These include how women feel during pregnancy, childbirth and subsequently, their experiences, associated factors, possible causal relationships, interventions that may support mental health and estimates of prevalence of mental health problems in various situations and groups.

Awareness and understanding of the impact of poor mental health on women is critical if their wellbeing is to be supported and maximised at this important time in their lives. Poor maternal mental health can affect subsequent child health and development [2] and is significantly associated with poor mental health among their partners [3]. Interventions and routine health and social care (including social support, screening, prevention and symptom management) may support better mental health, protecting or enabling women to adjust, function as mothers and partners and enjoy doing so.

## Issues of definition

Various constructs have been assessed by study authors in relation to the topic of maternal mental health. These include commonly investigated experiences such as depression, anxiety, ‘stress’ [4] and ‘distress’ [5, 6] as well as less commonly reported ‘wellbeing’ [7–9], ‘emotional disorders’ [10], symptoms of PTSD (Post Traumatic Stress Disorder) [11–13], suicidal ideation [14], mood instability [15], anger [16] and intolerance of uncertainty [17]. These experiences have been differentiated with regard to the ‘antenatal’ and ‘postnatal’ time periods, or both [18]. Some of the Collection papers refer specifically to the adverse experiences of pregnancy loss or bereavement [19], domestic violence [20] or being displaced during genocidal violence [21].

## Study design and measurement

Choosing appropriate methods and measures for a particular research study and purpose is critical, as are the comparison groups selected. In planning studies in which perinatal mental health is the primary focus or target, the outcomes are likely to be influenced by a range of potential confounding factors relating to individual history, demography and disadvantage, many of which need to described and taken into account.

However broad or narrow the terms used, clear definitions and descriptions are needed in designing research, with detailed information about the context and gaps in the existing literature, all of which will impact on interpretation, assessment of strengths, limitations and implications, as well as recommended further research. In addition to the variation in terminology used in the Collection papers, there is also diversity of measures reported in quantitative studies. A mental health related measure needs to be well developed and thoroughly validated, with clear definition and scope, to ensure clarity of findings, meaningful interpretation and reproducibility. This Collection includes large and small scale studies, cross-sectional studies [5, 16, 11], cohort studies [18, 22] and studies validating new screening or measurement tools [8, 13, 17].

While mostly cross-sectional studies were carried out, some described a randomized controlled trial (RCT) [9, 23, 24] or other longitudinal design [18]. A limited range of instruments was used, some of which were presented in validation papers. These included the Edinburgh Postnatal Depression Scale (EPDS), Spielberger State Trait Anxiety Inventory (STAI), Wellbeing in Pregnancy scale (WiP), Perceived Stress Scale (PSS) and the University of California, Los Angeles (UCLA) Loneliness Scale. New constructs such as intolerance of uncertainty [17] were also reported. The Collection also includes several qualitative studies; aiming to gain deeper understanding of individuals’ experiences; these generally take a bottom-up approach with broader research questions, utilising different methods such as thematic analysis [25], template analysis [19] and analysis informed by a critical realist approach [7].

One of the most useful methodologies to be employed in relation to a broad topic such as mental health is the process of systematic review. While no quantitative systematic reviews or meta-analyses were submitted, the Collection includes a scoping review reporting on the rapidly growing field of non-pharmacological interventions that aim to address both sleep and mood outcomes during the perinatal period [26]. Two systematic reviews of qualitative data are included; one summarises perceptions and attitudes around perinatal mental health in Bangladesh, India and Pakistan [27], while the other provides a qualitative evidence synthesis of data about women’s experiences and perceptions of anxiety and stress [28]. McNab et al. [29] present a ‘landscape analysis’ to assess the state of common perinatal mental disorders and strategies to address these in lower and middle income countries, and then call on the international community, government and health systems to act urgently to ensure that women everywhere have access to high-quality, respectful care for both their physical and mental wellbeing [30].

## Incidence, determinants and factors associated with poor and more satisfactory mental health

Studies on maternal mental health were carried out in a diversity of contexts and continents, including resource-poor settings. The papers included in the Collection were undertaken by researchers in more than twenty different countries in many parts of the world, ranging from Africa and South East Asia to those in more resourced settings such as Europe, the Middle East, Australia, Japan, China, and the USA.

Documenting prevalence was a primary objective in a number of the Collection papers [31–33], particularly in low and middle income country settings. Where factors were identified which predict poorer perinatal mental health, these were commonly associated with disadvantage and limited access to resources. Associations with possible predictors, including pregnancy acceptability [34], self-care [35], childhood trauma [36], alcohol and substance misuse [36] and infant crying [22] were explored. Other demographic and individual characteristics were recognised by some researchers as key in understanding some of the associations with poor, positive or improved mental health. These can include age, parity, childcare burden, education, engagement with work and employment, income, health, pregnancy complications, prior mental health and social support. While there may have been adjustment for some confounding or moderating variables, relatively few studies utilised cohort data collected at more than one time point, limiting the basis for modelling and exploring causal pathways in most cases.

Other methodological issues such as retrospective reporting of earlier mental health and wellbeing, timing of exposures and data collection (antenatally, postnatally, in later follow-up), and the way that groups are categorised all need to be considered carefully. Simply grouping participants by pregnancy or postnatal period is not sufficient as mental health is known to change over time.

Outcomes for infants can include medical data and psychosocial measures of infant health and behaviour, parent-child relationships and attachment.

## Interventions to support or improve maternal mental health

A limited number of studies reported interventions. These included interpersonal counselling, anxiety reduction, changes in healthcare provision, singing, group problem-solving, access to resources and the role of social support. Most were cross-sectional studies or utilised a short-term follow-up and were less likely to be in low-income settings. However, in a Ugandan feasibility study prior to a possible RCT, pregnant women in the second and third trimester were consecutively screened using the Luganda version of the 9-item Patient Health Questionnaire [37]. Those scoring at or above the cut-off and confirmed to have depression by a midwife were recruited into a treatment cohort and offered a psychological intervention in a stepped care fashion, with follow up at 3 and 6 months later. The apparent benefits of the intervention implemented in primary antenatal care by trained and supervised midwives in a real-world setting suggest that randomised studies are needed to confirm the efficacy of this kind of intervention and possible scaling up. Such studies are particularly important in low income settings, owing to poor maternal and infant outcomes [38]. Equally important, among women of low socio-economic position in the UK, a brief intervention showed promise in facilitating access to social support [39].

In one RCT, the aim was to investigate the impact of a mother-infant singing intervention in the first three months after birth on maternal well-being, depressive symptoms and bonding [9]. A causal pathway was evidenced with measures at several time points showing a significant effect on cortisol levels and mood, though not on symptoms of depression [9]. Another RCT involved a short-term psychological intervention, using cognitive behavioural therapy (CBT), resulting in anxiety reduction in pregnant women with positive screening for chromosomal disorders [23]. A significant reduction in pregnancy-related anxiety was demonstrated by Shen et al. [24], as well as reduced physical symptoms of anxiety among advanced multiparas, following needs-based education.

## Maternal mental health issues arising during the COVID-19 pandemic

Given the potential for distress, the experience of separation and isolation, changed or reduced maternity healthcare provision, and the serious health consequences for individuals and communities worldwide, it is no surprise that eleven papers are included in the Collection which consider maternal mental health during the COVID-19 pandemic. In many cases, these studies reported on prevalence and correlates of poor mental health [40–43] but are commonly hindered by lack of pre-pandemic data in the same populations. Interestingly, studies reporting a comparison of mental health of women pre- and during lockdowns, found no significant difference [4, 44, 45], or even reduced prevalence of anxiety during compared to pre-lockdown [46].

High-quality qualitative data on women’s experiences of becoming a mother during the pandemic [7, 19, 47] are particularly important for healthcare providers and policy makers to consider, given that the pandemic is by no means over and women-centred care remains a priority despite changes to healthcare access.

## The take home messages

As Collection Guest Editors, we would argue that maternal mental health and psychological functioning is a key aspect of health, impacting on the wellbeing and physical health of women and their families in multiple contexts and situations. The Collection papers reflect a diversity of approaches, settings and methods and can function as valuable resource for researchers, clinicians, policy makers and others working in this field.

Understanding and managing perinatal mental health is complex for healthcare providers and services; this should always be informed by high-quality evidence. The existing literature and any gaps in evidence, research questions asked, the way in which hypotheses are framed, and understanding of the diversity of women’s circumstances and experiences, are critical in planning and presenting high-quality research studies in this field. We recommend that:

- Though prevalence data may be an important cornerstone in planning and initiating further research, more emphasis on women’s needs is required.
- More RCTs, longitudinal studies, systematic reviews, meta-analyses and meta-syntheses, where possible, are required to summarise effective and feasible interventions to support evidence-based improvements in care, particularly in resource-constrained settings.
- Interventions should be designed with women, and authors reporting on these should acknowledge this contribution [26]. Fathers, partners and families should be considered where appropriate.
- Mechanisms of change (causal mechanisms, *how* interventions may affect outcomes) need to be suggested and supported by previous evidence, for all proposed interventions. Where possible, mediating and moderating factors should be explored using sophisticated statistical models [48].
- Language needs to be used cautiously and accurately, for example the ’impact’ of COVID-19 cannot be assessed in a cross-sectional study.

Research findings such as those presented in the Maternal Mental Health Collection can inform practice and clinical and community-based management of women’s mental health during the perinatal period. Quantitative studies are and should be considered alongside studies in which individual women’s voices are heard.
